# Gene variants associated with pediatric-onset erythromelalgia: Mendelian and rare-variant association analyses

**DOI:** 10.1097/PR9.0000000000001465

**Published:** 2026-08-03

**Authors:** Matthew C. Yonas, Don Daniel Ocay, Casie A. Genetti, Kimberly Lobo, Melissa Fernandes, Nicole Groussis, Meghan Halpin, Robert T. Wilder, Timothy W. Yu, Charles B. Berde, Catherine A. Brownstein

**Affiliations:** a MD Program, Harvard Medical School, Boston, MA, USA; b Harvard-MIT Program in Health Sciences and Technology, Harvard Medical School, Boston, MA, USA; c Department of Anesthesiology, Critical Care and Pain Medicine, Boston Children's Hospital, Boston, MA, USA; d Department of Anaesthesia, Harvard Medical School, Boston, MA, USA; e Manton Center for Orphan Disease Research, Boston Children's Hospital, Boston, MA, USA; f Department of Anesthesiology and Perioperative Medicine, Mayo Clinic, Rochester, MN, USA; g Division of Genetics and Genomics, Department of Pediatrics, Boston Children's Hospital, Boston, MA, USA; h Department of Pediatrics, Harvard Medical School, Boston, MA, USA

**Keywords:** Erythromelalgia, Pediatric, Gene, Variant, Mendelian analysis, Sequence kernel association test, Optimal

## Abstract

**Introduction:**

Erythromelalgia is a descriptive term for burning pain and erythema in distal extremities, often worsened by heat and improved by cold. Inherited erythromelalgia has been primarily linked to gain-of-function variants in *SCN9A*, encoding voltage-gated sodium channel NaV1.7. However, approximately 65% to 85% of patients with erythromelalgia do not have pathogenic *SCN9A* variants.

**Objectives:**

The objective of this study was to uncover and assess gene variants potentially associated with pediatric-onset erythromelalgia.

**Methods:**

With IRB approval and informed consent, probands and families with erythromelalgia underwent next-generation sequencing. A list of genes of interest was produced based on Mendelian inheritance models. Selected gene candidates were assessed using the Sequence Kernel Association Test-Optimal (SKAT-O).

**Results:**

Sixty-two probands with erythromelalgia and their relatives were included in Mendelian analysis, which identified variants in PR domain zinc finger protein 12 (*PRDM12*) and dihydropyrimidinase-like protein 2 (*DPYSL2*). In a targeted 12-gene rare-variant set analysis using SKAT-O, zinc finger homeobox protein 2 (*ZFHX2*) showed evidence of association (*P* = 6.9 × 10^−4^), surpassing Bonferroni correction for 12 tests (α = 4.17 × 10^−3^), whereas *KIF1B* showed only a nominal association signal (*P* = 0.03) that did not survive multiple-testing correction.

**Conclusion:**

Genes associated with both increased and decreased pain sensitivity are of considerable interest for elucidating pain mechanisms and analgesic development. As rare variants in *PRDM12* and *DPYSL2* were identified in a pediatric erythromelalgia cohort and a gene-based rare-variant association signal for *ZFHX2* was identified, replication and functional validation are needed.

## Abstract 2

Supplemental Digital Content is Available in the Text.

We report variants in PRDM12 and DPYSL2 in a pediatric erythromelalgia cohort and a gene-based rare-variant association signal for ZFHX2 in a hypothesis-driven test set.

## 1. Introduction

Erythromelalgia is a rare pain disorder^2,21,28,41,47^ characterized by episodes of erythema, burning pain, and elevated temperature in the distal extremities which are usually exacerbated by heat and alleviated with cooling. Despite its rarity, erythromelalgia is a debilitating disorder with substantial clinical impact, necessitating further research into its pathophysiological underpinnings. Erythromelalgia is mainly classified based on its origin as either primary or secondary. Primary erythromelalgia may be inherited or arise spontaneously, with inherited cases frequently linked to gain-of-function variants in *SCN9A*, which encodes the NaV1.7 voltage-gated sodium channel.^12^ Gain-of-function variants in *SCN9A* lead to hyperexcitability of nociceptive neurons by lowering the action potential threshold, thereby amplifying pain perception.^15^ However, most patients (65%–85%) diagnosed with erythromelalgia lack pathogenic *SCN9A* variants.^8,40,48^ Secondary erythromelalgia, by contrast, can be associated with underlying conditions such as autoimmune and vascular diseases, and myeloproliferative disorders.^28^

Erythromelalgia is relatively understudied, and mechanisms driving disease pathogenesis remain incompletely understood. While primary erythromelalgia has been linked to an ion channelopathy,^53^ emerging evidence suggests that additional genes may be involved. Variants in *SCN9A* are also implicated in other chronic pain conditions, including congenital insensitivity to pain^11^ and small fiber neuropathy.^19^ Beyond *SCN9A*, variants in ion channel-encoding genes including *SCN10A*, *SCN11A*, and *TRPA1* have been associated with clinical phenotypes resembling erythromelalgia.^3,28,59^ Further exploration of potential genetic contributors may yield targeted therapies. In cases of secondary erythromelalgia associated with thrombocytosis and *JAK2* variants, aspirin provided symptomatic relief.^37^ However, treatment options for primary erythromelalgia remain limited.^36^ Sodium channel modulators are under investigation as potential long-term therapeutic options.^1^

Genome-wide association studies have been instrumental in identifying genetic variants associated with various diseases, but their utility in rare disorders such as erythromelalgia is limited by small sample sizes and an inability to capture the full spectrum of rare variant effects. The sequence kernel association test-optimal (SKAT-O) provides a powerful alternative for rare variant association studies, as it aggregates genetic variants across a region to evaluate their collective associations while accounting for both protective and deleterious effects.^32,55^ This approach is particularly well-suited for studying erythromelalgia, where rare variants in multiple genes may contribute to disease susceptibility.

Despite advances in understanding *SCN9A*-associated erythromelalgia, the genetic landscape of the disorder remains incompletely defined. Our previous clinical characterization of pediatric erythromelalgia at Boston Children's Hospital revealed that only 3 of 42 patients harbored confirmed likely pathogenic *SCN9A* variants,^49^ underscoring the need for broader genetic investigations. In this study, we hypothesized that deleterious variants affecting non-*SCN9A* pathways contribute to erythromelalgia pathogenesis in distinct patient subsets. Our objectives were to perform Mendelian analyses to identify candidate genes in erythromelalgia patients and complete SKAT-O analyses to assess the association of specific rare variants with the phenotype. We anticipated that this approach would uncover genetic contributors to erythromelalgia, thereby expanding the current understanding of its molecular basis and informing future therapeutic strategies.

## 2. Methods

### 2.1. Patient recruitment, phenotyping, and diagnosis

A collaborative multicenter pediatric erythromelalgia consortium spanning the United States, Canada, the United Kingdom, and Australia was established to investigate the clinical features, natural history, treatment responses, mechanisms, and genetic underpinnings of early-onset erythromelalgia. From March 2023 onward, individuals with a clinical diagnosis of erythromelalgia were identified through (1) referrals from consortium physicians, or (2) self-referral in response to research recruitment materials posted in online support group forums. Eligibility criteria for probands included childhood onset of erythromelalgia (before 18 years of age) as previously described ^40^ and adapted from Brown.^7^ Exclusion criteria included (1) inability to provide informed consent or comply with the study protocol, (2) incomplete clinical documentation preventing a definitive diagnosis of erythromelalgia, or (3) inability to confirm a clinical diagnosis of erythromelalgia on consultation with the referring physician or a physician of our team (C.B.B.).

To increase the breadth of identifying variants associated with erythromelalgia and due to the spectrum of hypersensitivity and hyposensitivity associated with *SCN9A* variants, probands with mixed clinical phenotypes with features overlapping with erythromelalgia and variations in pain sensitivity were also recruited. Probands could be either adults or children, and all were enrolled following informed consent under an IRB-approved protocol at Boston Children's Hospital (IRB ID: *10-02-0053*): The Manton Center for Orphan Disease Research Gene Discovery Core.

After enrollment, a three- to four-generation pedigree was obtained for each proband, with a focus on the presence and pattern of pain symptoms consistent with erythromelalgia. Additional family members were recruited using a tiered priority system based on the number of affected relatives and the strength of the familial pain phenotype.

### 2.2. Sample collection and next-generation sequencing

Blood and/or saliva samples were collected from probands and enrolled family members using standard protocols. Whole-exome sequencing or whole-genome sequencing on the Illumina platform was performed at multiple centers—including the Broad Institute (Cambridge, MA), GeneDx (Stamford, CT), Psomagen (Rockville, MD), DeCode (Reykjavik, Iceland), Ambry (Aliso Viejo, CA), and Claritas Genomics (Waltham, MA).

### 2.3. Variant annotation and filtering

After next-generation sequencing, all variants were annotated for their predicted effects on protein function using the Codified Genomics platform (San Diego, CA). To identify potential disease-causing variants, a minor allele frequency (MAF) threshold of 0.005 was applied for putative autosomal dominant variants and 0.01 for autosomal recessive variants. Public databases including gnomAD, dbSNP, and the 1000 Genomes Project, in addition to 2672 internal control sequences (healthy parents of individuals with suspected Mendelian disorders), were used to estimate allele frequencies. PolyPhen and other in silico tools were used to predict pathogenicity.

All samples were also processed with the Genome Analysis Toolkit according to best practices,^43^ and variant call format data were analyzed through Gregor, an HPO-based blinded strategy within the GeneDx Browser (formerly Genuity Science, version 2.0.1, Cambridge, MA). Variants underwent the same MAF cutoffs (0.005 for autosomal dominant, 0.01 for autosomal recessive), and only variants with high or moderate impact, as predicted by Variant Effect Predictor,^25^ were considered. GeneDx automatically screened variants against ClinVar, HGMD, and OMIM.

### 2.4. Gene prioritization Through Mendelian analysis

Candidate variants in each proband were aggregated into a preliminary gene list. Genes with established gene-disease contexts were then evaluated using automated American College of Medical Genetics sequence variant classification criteria^42^ in VarSome (https://varsome.com) and Franklin by Genoox (https://franklin.genoox.com) and were manually reviewed to confirm clinical interpretation. Variants classified as “Variant of Uncertain Significance” (VUS), “Likely Pathogenic” (LP), or “Pathogenic” were cross-checked in ClinVar.

Genes without validity for gene–disease associations with pain syndromes were assessed using a set criteria: First, the gene was classified as having a definitive, strong, moderate, limited, disputed, refuted, or no association with erythromelalgia or a syndrome involving a pain phenotype (eg, disorders involving pain, neuropathy, ion channels, receptors, neurotransmitters, nervous system function, or vascular tone regulation phenotypes). The biological relevance of the candidate genes was queried using a literature search and assessment of publicly available databases including the Allen Brain Atlas,^29^ ClinGen,^45^ and String.^50^ Model organism databases such as zfin^5^ were also used to obtain evidence for or against the variant/gene/interval's hypothesized involvement in the phenotype. Genome-wide association study (GWAS) data were examined to determine whether the gene is a candidate for this phenotype or related phenotypes in the GWAS catalog and published literature.^9,33^ Each gene was assessed by 2 or more individuals. Any disagreements in categorization were resolved in a meeting consisting of physicians, geneticists, genetic counselors, and research assistants.

### 2.5. Biological plausibility prioritization scoring

To systematically identify and rank the most promising hypothesis-generating candidate genes, participant demographic and clinical data were first integrated with the curated gene library using REDCap (Boston Children's Hospital). Each gene was scored using a predefined set of criteria intended for internal candidate prioritization (not a validated pathogenicity or gene-disease validity scoring system). Criteria included (1) detection of variants in the same gene in multiple probands; (2) evidence of variant segregation among affected relatives; (3) determined to have a definitive, strong, moderate, limited, or disputed link to erythromelalgia or disorders involving pain, neuropathy, ion channels, receptors, neurotransmitters, nervous system function, or vascular tone regulation phenotypes; (4) presence in individuals with pain insensitivity (in cohort or literature) in addition to phenotypes/disorders listed in #3; and (5) potential pathogenicity classification of all variants within the gene (CADD score over 18 or SpliceAI over 0.5) (Table 1).

**Table 1:** Heuristic biological plausibility rubric used to prioritize candidate genes for follow-up.

Ranking criterion	Point value if “yes”
Variants in this gene were identified in more than one proband	+1
Variants segregate with disease status	+1
Gene variants were present in probands with erythromelalgia and probands with congenital insensitivity to pain	+1
The candidate gene has a definitive, strong, moderate, limited, or disputed association with a pain, neuropathy, ion channels, receptors, neurotransmitters, nervous system function, or vascular tone regulation disorder or phenotype	+1
Potential impact of variants on protein: All variant CADD scores 18+ or splice AI scores 0.5+	+1

Each gene is assigned 1 point per criterion met (maximum 5 points). Total scores are used only to rank candidates for downstream analyses and are not intended as a validated pathogenicity or gene–disease validity framework.

CADD, combined annotation dependent depletion.

Genes meeting 3 or more criteria were labeled as strong candidates, those satisfying 2 criteria were moderate candidates, and genes with fewer than 2 criteria were weak candidates. Subsequent analyses concentrated on strong and moderate candidates, incorporating additional literature reviews on genes implicated in erythromelalgia, insensitivity to pain, and neuropathy. Because rare variants are statistically challenging to interpret, the study ultimately focused on this refined, high-priority gene set.

### 2.6. Statistical analysis

SKAT-O in the GeneDx implementation (version: 2.4.1, 2022-09-21, Package version: 8.11.1, Git SHA: 854cb277) was used to examine the association of rare variants in candidate genes among probands with erythromelalgia. The SKAT-O characteristics were: model type (Dichotomous [Logistic/Binary]), kernel/weights (linear.weighted, Beta [1,25], and no covariates were used). The rare-variant set definition was coding only and variants were collapsed by gene boundary only.

Cases were compared against matched controls (N = 62) obtained from the Manton Center for Orphan Disease Research at Boston Children's Hospital; controls were unaffected parents of patients with unrelated Mendelian disorders. Matching was performed based on sex, race, ethnicity, and sequencing platform.

Preprocessing steps removed cases not sequenced at GeneDx, those with a known diagnostic variant, and cases with poor sequencing coverage or low yield (ie, fewer than 80% of expected variants called). This filtering left 35 affected individuals (18 female, 17 male) and 62 controls (32 female, 30 male). Variants with MAF above 0.01 were excluded, and only high/moderate impact variants (stop gained/lost, frameshift, splice donor/acceptor, missense) were included. SKAT-O analyses on 12 genes were performed in the GeneDx browser. A Bonferroni correction was applied to determine the statistical level of significance.

To control for potential batch effects and systematic bias, 2 sets of “housekeeping” genes were selected from a published database^18^ using a random number generator. Genes associated with pain pathways or neuropathic pain were excluded. These “housekeeping” genes were analyzed in the same manner described above.

## 3. Results

### 3.1. Mendelian analysis

At the time of analysis (June 2024), Mendelian analysis was performed on 62 probands with erythromelalgia and 145 additional relatives; 31 of these family members had an erythromelalgia diagnosis and 114 were unaffected relatives. Seven probands did not have any relatives enroll.

Assessment of genes with pain syndrome validity identified *SCN9A* variants in 6 probands (Table 2, Supplementary Fig. 1, http://links.lww.com/PR9/A419). NM_001365536.1(SCN9A):c.2606T>A (p.Leu869His (SCV003524824)) and c.1198G>A (p.Val400Met (SCV000769590)), both pathogenic variants previously reported for erythromelalgia,^40^ were identified in a maternally inherited individual (proband #82) and de novo in the other individual (proband #80), respectively. The previously reported pathogenic variant NM_001365536.1(SCN9A):c.2600G>A (p.Gly867Asp (SCV000293491)) was identified in a maternally inherited individual (proband #7) and their affected sibling. This proband (#7) also presented with short stature, which has been associated with a nearby SCN9A variant (2567G>C; p.Gly856Arg).^26,51^ NM_001365536.1(SCN9A):c.1754G>A (p.Arg585Lys, previously reported as a VUS (SCV006072408)) and NM_001365536.1(SCN9A):c.706G>T (p.Gly236Trp, previously reported as Likely Pathogenic (SCV005857206)) were identified in maternally inherited individuals (proband #31 and proband #27, respectively), and the proband's (#27) affected sibling. NM_001365536.1(SCN9A):c.2287A>G (p.Met763Val, previously reported as a VUS (SCV001387399)) was identified in a maternally inherited individual (proband #32) and their affected mother.

**Table 2:** SCN9A variants identified in probands with erythromelalgia in the Mendelian analysis (ClinVar accessed January 1, 2026).

Variant	Protein change	ACMG criteria classification	ClinVar reported classification	ClinVar submission accession
NM_001365536.1:c.2606T>A	p.Leu869His	P	P	SCV003524824
NM_001365536.1:c.1198G>A	p.Val400Met	P	P	SCV000769590
NM_001365536.1:c.2600G>A	p.Gly867Asp	P	LP	SCV000293491
NM_001365536.1:c.1754G>A	p.Arg585Lys	VUS	VUS	SCV006072408
NM_001365536.1:c.706G>T	p.Gly236Trp	LP	LP	SCV005857206
NM_001365536.1:c.2287A>G	p.Met763Val	VUS	VUS	SCV001387399

Variants are listed using HGVS nomenclature (transcript NM_001365536.1) with the corresponding protein change. The table reports the ACMG/AMP classification assigned in this established gene–disease context and the ClinVar-reported classification along with the ClinVar submission accession number.

ACMG, American College of Medical Genetics; LP, likely pathogenic; P, pathogenic; VUS, variant of uncertain significance.

Analysis revealed 180 unique candidate genes with a categorized (eg, Definitive/Strong/Moderate/Limited/Disputed) link to erythromelalgia or disorders involving pain, neuropathy, ion channels, receptors, neurotransmitters, nervous system function, or vascular tone regulation phenotypes (Table 3). As part of the analysis, we cross-referenced candidate genes/variant-bearing genes against GWAS pain loci summarized in Li et al.^33^; 5 genes overlapped (Supplemental Table 1, http://links.lww.com/PR9/A419). The biological plausibility prioritization scoring divided the genes into 29 “strong” candidate genes, 78 “moderate” candidate genes, and 73 “weak” candidate genes (Table 3).

**Table 3:** Biological plausibility categorization of candidate genes identified from Mendelian analysis of 62 probands with erythromelalgia.

Strong	Moderate			Weak	
*ABCA1* *ADAMTS2* *ALPK3* *CLCN1* *COL6A3* *COL7A1* *DPYSL2* *FLG* *GJB2* *KCNN1* *KCNU1* KIF1B *LAMB3* *MINK1* *NBEAL1* *PRDM12* *RYR1* *SCN10A* *SCN2A* *SCN5A* *SCN9A* *SHANK3* *SPG7* *SYNE2* *TNXB* *TRPV4* *TTR* *WNT10A* *ZFHX2*	*ABCC2* *ACCN2* *ACOT9* *AKAP6* *ARID1A* *ATXN3* *BSCL2* *CACNA1A* *CACNA1C* *CALR* *CDH2* *CDKN2C* *CHL1* *COL11A2* *COL5A2* *CPS1* *CRLF1* *CRMP1* *CYFIP1* *CYP27A1* *DNMT1* *DSG4* *DST* *EFCAB4B* *EPHA4* *F11* *FAT2* *GAD1* *GLA* *GRM8* *HERC2* *IAH1* *IGHMBP2* *KCNH5* *KCNH6*	*KCTD1* *KDM6B* *KIF26A* *KIF5A* *KL* *LAMC1* *LBR* *LMX1B* *LPHN1* *LRRFIP1* *LRRK2* *LRSAM1* *LYNX1-SLURP2* *MEF2B* *MRGPRX1* *MSH2* *NFKB1* *NLRP12* *NOD2* *NTSR1* *NUMA1* *P2RX7* *PADI4* *PDZD2* *PREP* *PTPRZ1* *RERE* *RNF167* *SCN1B* *SCN3A* *SCN7A* *SCN8A* *SELENON* *SERPINA3* *SPAST*	*STAU1* *SYN3* *TFRC* *TREX1* *TRIM71* *TRPM5* *TRPV1* *TTN*	*ACAN* *ADAMTS10* *ATL1* *ATL3* *C1QC* *CACNA1B* *CBLN1* *CCDC57* *CCR1* *CELF1* *CLEC11A* *CNTNAP2* *COL12A1* *COQ2* *CTNNB1* *CXorf51A* *DSP* *EPRS* *ERV3-1* *F8* *FAM135B* *FAM86DP* *FLNC* *FOXO4* *GDAP1* *GRM7* *GTPBP4* *HAUS7* *HERC4* *HTR2A* *IMMP2L* *KCNG2* *KIF24* *KIF26B* *KMT2C* *LPL* *MBTD1*	*MEFV* *MRGPRX2* *MTMR7* *MYH7* *MYOT* *NAV2* *NBPF1* *NDUFB11* *NLGN4X* *NTNG1* *PAPSS2* *PFKB1* *PHKA1* *PLXNA2* *POFUT2* *POLE* *POLG* *POLM* *PRODH* *RNF170* *RPS17* *RREB1* *SH3TC2* *SLC4A1* *SLC6A4* *SMC1A* *SNX24* *STAG2* *STOM* *TPCN1* *Tra2b* *TRIM2* *TRPC6* *UGT2B15* *UPP1* *ZBTB10*

A total of 180 unique candidate genes were compiled and then prioritized using the rubric in Table 1. Genes are grouped into heuristic categories based on the number of criteria met: Strong (≥3 criteria), Moderate (2 criteria), and Weak (<2 criteria). These categories are intended to highlight genes for hypothesis generation and follow-up and do not, on their own, establish causality or a definitive gene–disease relationship.

### 3.2. Notable cases with candidate genes

The gene PR domain zinc finger protein 12 (*PRDM12*) has an associated biallelic LOF congenital insensitivity to pain phenotype.^10^ We identified heterozygous missense *PRDM12* variants in patients with erythromelalgia/hypersensitivity that remain hypothesis-generating pending functional validation (Supplementary Fig. 1, http://links.lww.com/PR9/A419). A *PRDM12* variant (chr9:130668264 NM_021619.3:c.521A>G), resulting in p.Asn174Ser in exon 3, was identified in a 39-year old erythromelalgia proband (#50) and her 12-year old erythromelalgia-affected daughter. This variant has not been identified in gnomAD 4.1.0 (accessed January 6, 2026) and genomic constraint of the surrounding 1 kb region (chr9:130668000–130669000) is Z = 4.02 (o/e 0.76). Missense constraint of the *PRDM12* gene is Z = 0.25. The proband had an onset of distal extremity burning pain, erythema, and swelling, worsened by heat, around the age of 16 years with persistence of these signs and symptoms for more than 2 decades. The proband had extensive neurologic and rheumatologic evaluation that did not identify specific/secondary disorders. Electrodiagnostic studies were normal and skin biopsies were interpreted as normal skin neurite density in one location and lower limit of normal for that laboratory in another location. The proband's daughter had an onset of distal extremity burning pain, erythema, swelling, and heat intolerance around the age of 10 years. The proband (#50) has an unaffected and healthy brother, and a paternal half-sister in good health.

We also identified a different *PRDM12* variant in proband #49, a heterozygous 12 base pair inframe deletion within exon 5 (chr9:130681606-130681617). The proband was a 10-year old girl with an onset of erythromelalgia at the age of 7 years. She presented with burning pain, redness, and blistering on the ears, hands, and feet, with the right ear particularly affected, which underwent surgery in the past. Despite the burning sensation, proband #49 reports cold hands and feet. The proband reports that cooling packs and gabapentin are beneficial, and that none of the immediate family members are affected.

### 3.3. Mendelian analysis for patients with features overlapping with erythromelalgia and variations in pain sensitivity

We also identified variants in probands in our cohort with *PRDM12* variants and abnormal pain processing (hyper- and hyposensitivity, Supplementary Fig. 1, http://links.lww.com/PR9/A419). A 21-year-old female proband (#16) with hyposensitivity to heat with a history of inadvertently burning herself had a maternally inherited 12-kb duplication containing exons 1-3 of *PRDM12* (dup:chr9:130659613-130671613). Of note, despite the hyposensitivity, the proband also reported widespread neuropathic pain, fibromyalgia, headaches, and dysautonomia whose onset was at the age of 2 years. The proband's family history includes a mother and maternal grandmother with fibromyalgia, and the mother also has a history of migraines, hypokalemia, and chronic fatigue. This proband has a maternal aunt with lupus affecting the central nervous system, a father and maternal grandfather with rheumatoid arthritis, and a paternal grandmother with multiple sclerosis.

A 2-year-old female proband (#95) with insensitivity to pain, with multiple instances of injury including cutting her fingers, biting her tongue, and nose scratching until bleeding that have not resulted in irritability, was identified to have a homozygous 18/18 alanine repeat expansion in *PRDM12* (NM_021619.3(PRDM12):c.1041CGC[18/18]), known to result in midfacial toddler excoriation syndrome.^44^ At age 3, she developed episodic erythema in her feet and hands, increased scratching of her toes and fingers, and a preference for cold and aversion to heat. The proband has 5 healthy siblings, and no other family history of pain insensitivity or hypersensitivity.

Another hypothesis-generating candidate gene that we identified through Mendelian analysis (Supplementary Fig. 1, http://links.lww.com/PR9/A419) as a potential candidate for erythromelalgia was dihydropyrimidinase-like protein 2 (*DPYSL2*), also known as the collapsin response mediator protein 2 (*CRMP2*). The proband (#52) is an 8-year-old girl with a complex mixture of abnormal temperature regulation, partial hyposensitivity to pain, and episodic erythema of the hands and feet evoked by heat, but also cyanosis evoked by cold. The proband also has 2 younger identical twin sisters (7 years old) with a similar phenotype. We identified a missense variant in *DPYSL2* in all 3 siblings (chr8:26647762 NM_001197293.3:c.1558G>A, p.Asp520Asn). All 3 displayed some degree of mild developmental delay and mild autism. Their family history includes a mother with a history of developmental issues, bipolar disorder, substance misuse, and some difficulties with balance, a father with schizophrenia, and an uncle with autism. All 3 siblings underwent a modified quantitative sensory testing battery with the proband displaying primarily a somatosensory loss profile, particularly to mechanical and vibration detection, and hypoalgesia to heat. Among the affected the twin sisters, one displayed a mechanical hyperalgesia and one had a normal somatosensory profile.

### 3.4. Sequence kernel association test-optimal analysis

To evaluate aggregate rare-variant associations within a set of 12 genes selected from our candidate gene list (Table 4), we performed gene-based rare-variant set testing using SKAT-O within the GeneDx browser (v2.0.1) restricted to pediatric-onset erythromelalgia cases sequenced at GeneDx and matched controls (Table 4, Supplementary Table 2, http://links.lww.com/PR9/A419). Analysis of housekeeping genes did not reveal significant differences between cases and controls. *ZFHX2* demonstrated evidence of association (*P* = 6.9 × 10^−4^), which remained significant after correction for 12 tests (Bonferroni α = 0.05/12 ≈ 4.17 × 10^−3^). *KIF1B* showed a nominal association signal (*P* = 0.03) but did not survive multiple-testing correction. These results were used to prioritize candidate genes for follow-up rather than to infer precise effect sizes, which are unstable in small cohorts with sparse rare-variant counts.^54^

**Table 4:** Rare-variant gene-based association testing (sequence kernel association test-optimal) for 12 high-priority candidate genes.

Gene	Total score	>1 proband?	Identified in a proband with EM in our cohort?	Shared gene between a proband with EM and with CIP in our cohort?	Definitive, strong, moderate, limited, or disputed association with pain or neuropathy disorder/s?	Variants segregate with disease?	SKAT-O markers	*P* (uncorrected)	Significant after Bonferroni correction for 12 tests (≈4.17 × 10^−3^)
*SCN9A*	5	Yes	Yes	Yes	Yes^59^	Yes	9	0.06	
*PRDM12*	5	Yes	Yes	Yes	Yes^44^	Yes	7	0.35	
*ABCA1*	4	Yes	Yes	No	Yes^46^	Yes	10	0.69	
*DPYSL2*	4	Yes	Yes	No	Yes^35^	Yes	4	0.10	
*SCN2A*	4	Yes	Yes	No	Yes^52^	Yes	3	N/A	
*SCN5A*	4	Yes	Yes	No	Yes^34^	Yes	3	N/A	
*SCN10A*	4	Yes	Yes	No	Yes^20^	Yes	5	0.65	
*SHANK3*	4	Yes	Yes	No	Yes^24^	Yes	5	0.59	
*TRPV4*	4	Yes	Yes	No	Yes^27^	Yes	7	0.42	
*ZFHX2*	4	Yes	Yes	No	Yes^23^	Yes	7	6.9 × 10^−^^4^	Yes
*KIF1B*	3	No	Yes	No	Yes^56^	Yes	8	0.03	No
*WNT10A*	3	No	Yes	No	Yes^30^	Yes	5	0.21	

Twelve genes were selected from the “Strong” and “Moderate” categories in Table 3 for hypothesis-generating rare-variant association testing. For each gene, the table summarizes the Table 1 total score, supporting evidence flags (eg, recurrence across probands, segregation, EM/CIP overlap), the number of qualifying variants included (SKAT-O markers), and the uncorrected SKAT-O *P*-value. SKAT-O was run using a logistic/binary model with Beta (1,25) variant weighting, no covariates, and a coding-variant set definition collapsed by gene; analyses compared 35 affected individuals with 62 matched controls. Statistical significance after multiple testing is assessed using a Bonferroni threshold of ∼4.17 × 10^−3^ (0.05/12 tests). “N/A” indicates that a *P*-value could not be calculated due to insufficient qualifying variants/markers under the analysis settings.

CIP, congenital insensitivity to pain; EM, erythromelalgia; SKAT-O, sequence kernel association test-optimal.

## 4. Discussion

While erythromelalgia has been most strongly linked to gain-of-function variants in *SCN9A*, many cases remain genetically unexplained, highlighting the likelihood that other genes may contribute to its etiology.

Through Mendelian analysis, *PRDM12* emerged as a gene of interest for erythromelalgia. *PRDM12* is a member of the PRDM (PR domain-containing) family of transcriptional regulators involved in cell fate decisions.^39^ These proteins are often associated with histone methyltransferase activity, although not all PRDM members have catalytic function. *PRDM12* is expressed during the development of the sensory nervous system and plays a critical role in specifying nociceptive neurons. Biallelic loss-of-function variants in *PRDM12* during development are a cause of congenital insensitivity to pain in humans.^10,13,31,58^ Current evidence suggests *PRDM12* modulates the histone methylation landscape important for sensory neuron development.^13,31^ Therefore, multiple hypotheses emerge for the possible role of *PRDM12* in the pathogenesis of erythromelalgia. Genes regulating excitability or nociceptor development, such as *PRDM12*, could generate a phenotype similar to erythromelalgia if they become dysregulated in a way that increases neuronal firing.^15^ In addition, it is possible that pathogenic variants in *PRDM12* that do not completely abolish function, but instead alter regulatory specificity, might lead to upregulation of excitatory genes (eg, sodium channels or other pronociceptive factors) or downregulation of inhibitory components. The result could be a net hyperexcitability of sensory neurons akin to the mechanism seen in erythromelalgia. Finally, as *PRDM12* helps specify nociceptor identity,^39^ a variant that partially misdirects this specification could possibly produce nociceptors with aberrant ion channel expression profiles, predisposing to spontaneous or exaggerated firing. The available evidence is currently insufficient to determine the role of this variant identified in erythromelalgia. Future functional work will clarify this gene's importance to pain sensation.

Another gene of interest for erythromelalgia that emerged through Mendelian analysis was *DPYSL2* (also known as *CRMP2*). *DPYSL2* plays an important role in early stages of brain development, particularly in coordinating axonal elongation and neurite outgrowth through its interaction with tubulin and actin in the brain. Studies on the genetic deletion of *DPYSL2* in mice have shown a direct link with impairment of dendritic patterning and a reduction in synaptic density contributing to the pathophysiology of neurodevelopmental disorders which fit the familial phenotype of the proband (#52) and their 2 sisters in our cohort.^14,57,60^ *DPYSL* mRNAs are found in glial cells in addition to excitatory and inhibitory neurons, suggesting a potential role in inflammatory pathways.^38^ There are several hypotheses in which *DPYSL2* variants may play a role in the pathophysiology of erythromelalgia. *DPYSL2*, in presynaptic terminals, regulates trafficking to the voltage-gated sodium channels 1.7 (Na_V_1.7) and N-type voltage-gated calcium channel (Ca_V_2.2).^6,17^ Therefore, upregulation of *DPYSL2* may lead to inflammatory and neuropathic pain. Moreover, *DPYSL2* may play a role in the effect of inflammation on neurotransmission of vascular smooth muscle cells, through its interaction with adrenoceptor α 1D (*ADRA1D*) or calcium voltage-gated channel subunit α1 S (*CACNA1S*).^22^ Dysregulation in *DPYSL2* may play a role in the abnormalities in thermoregulation present in proband #52.

In our targeted rare-variant set analysis, we used SKAT-O to test whether aggregated rare coding variation across a hypothesis-driven set of 12 high-priority genes differed between pediatric-onset erythromelalgia cases and matched controls. *ZFHX2* showed evidence of association (*P* = 6.9 × 10^−4^) and remained significant after multiple-testing correction. ZFHX2 encodes Zinc Finger Homeobox 2, a transcription factor highly expressed in dorsal root ganglion sensory neurons and implicated in pain regulation.^23^ A pathogenic missense variant in ZFHX2 (14:23993413C>T, p.Arg1913Lys) has been shown to cause Marsili syndrome, characterized by selective insensitivity to pain.^23^ Consistent with this role, ZFHX2 loss-of-function is associated with reduced pain sensitivity, and mouse models demonstrate altered responses to mechanical and thermal stimuli after gene disruption. While knockout mice show attenuated nociception and hypersensitivity to noxious heat, transgenic models carrying the p.Arg1907Lys variant display reduced heat sensitivity, highlighting allele- and context-specific effects.^23^ Although the variants identified in this cohort do not localize to the ZFHX2 homeodomain, prior human and animal studies support a broader role for ZFHX2 in modulating pain perception. We therefore hypothesize that nonhomeodomain variants may influence pain sensitivity through effects on protein stability or expression, potentially involving antisense-mediated regulatory mechanisms, although the precise molecular consequences remain to be determined.

### 4.1. Limitations

A key limitation of this study is the small sample size, which reduced the statistical power necessary to detect potential associations between rare variants and erythromelalgia. Current human gene-disease validity is limited; our results provide candidate-level evidence only. Another limitation is the lack of functional work in the identified variants of our probands to ascertain their association with erythromelalgia.

In addition, the absence of certain genetic markers in our data set restricted our ability to capture the full spectrum of potentially pathogenic variants. Our rare-variant analyses prioritized coding variants with predicted high or moderate impact, but this approach may miss regulatory variants, promoter or enhancer variants, untranslated-region variants, deep intronic splice-altering variants, and other changes that affect mRNA abundance or isoform expression. Future research should prioritize larger, more diverse cohorts; integrate comprehensive sequencing methods (eg, long-read or whole-genome sequencing); single-cell RNAseq and transcriptomics for supportive biological plausibility evidence such as expression in the dorsal root ganglion^4^; and additional blinded gene prioritization to refine candidate gene discovery.

Rare-variant kernel tests such as SKAT-O rely on asymptotic results and numerical evaluation of mixture chi-square tail probabilities, and the accuracy of *P*-values can depend on the computational method used. Prior work has shown that commonly used implementations can yield conservative or inflated type I error in some settings, including situations in which numerical routines fail to converge or where approximations to the null distribution are anticonservative at very small significance levels.^54^ These considerations are particularly relevant for modest sample sizes and sparse rare-variant counts, where calibration of extreme *P*-values can be challenging. The SKAT-O findings should be interpreted cautiously and considered hypothesis-generating pending replication in larger, harmonized data sets and analyses that incorporate resampling-based calibration.

Importantly, there is no consensus on the diagnostic criteria for erythromelalgia.^7,16^ Heterogeneity in the diagnostic process may have been introduced into our sample enrollment, but all probands met both the original Brown criteria^7^ as well as our recently proposed adaptation of Brown's criteria for pediatric erythromelalgia.^40^

## 5. Conclusions

We identified *PRDM12*, *DPYSL2*, and *ZFHX2* as potential candidate genes in erythromelalgia etiology warranting further study. *PRDM12* and ZFHX2 variants have previously been associated with pain insensitivity, but the hypothesis is that variants in these genes may produce increased spontaneous pain responses within the erythromelalgia spectrum. *DPYSL2* has been shown to be involved in neuroinflammatory pathways, making it a gene of considerable interest. These findings extend our current understanding of erythromelalgia and provide avenues for future work aimed at decoding its genetic architecture. Through continued gene discovery efforts and advanced bioinformatics approaches, the elucidation of erythromelalgia's pathophysiology may reveal targets for precision analgesic therapies and ultimately improve clinical outcomes for affected individuals.

## Disclosures

The authors have no conflict of interest to declare.

## Supplemental digital content

Supplemental digital content associated with this article can be found online at ://links.lww.com/PR9/A419.
