# Supplementary material for: Gene variants associated with pediatric-onset erythromelalgia: Mendelian and rare-variant association analyses
Source: Pain Rep. 2026 Aug 3;11(5):e1465. doi: 10.1097/PR9.0000000000001465 (PMC13441107; doi:10.1097/PR9.0000000000001465)
Supplement: Supplementary file 1 [file painreports-11-e1465-s001.pdf]

Supplementary Figure 1. Pedigrees of reported cases and family members, in order of appearance, with variants in A) *SCN9A*, B) *PRDM12*, or C) *DPYSL2/CRMP2*

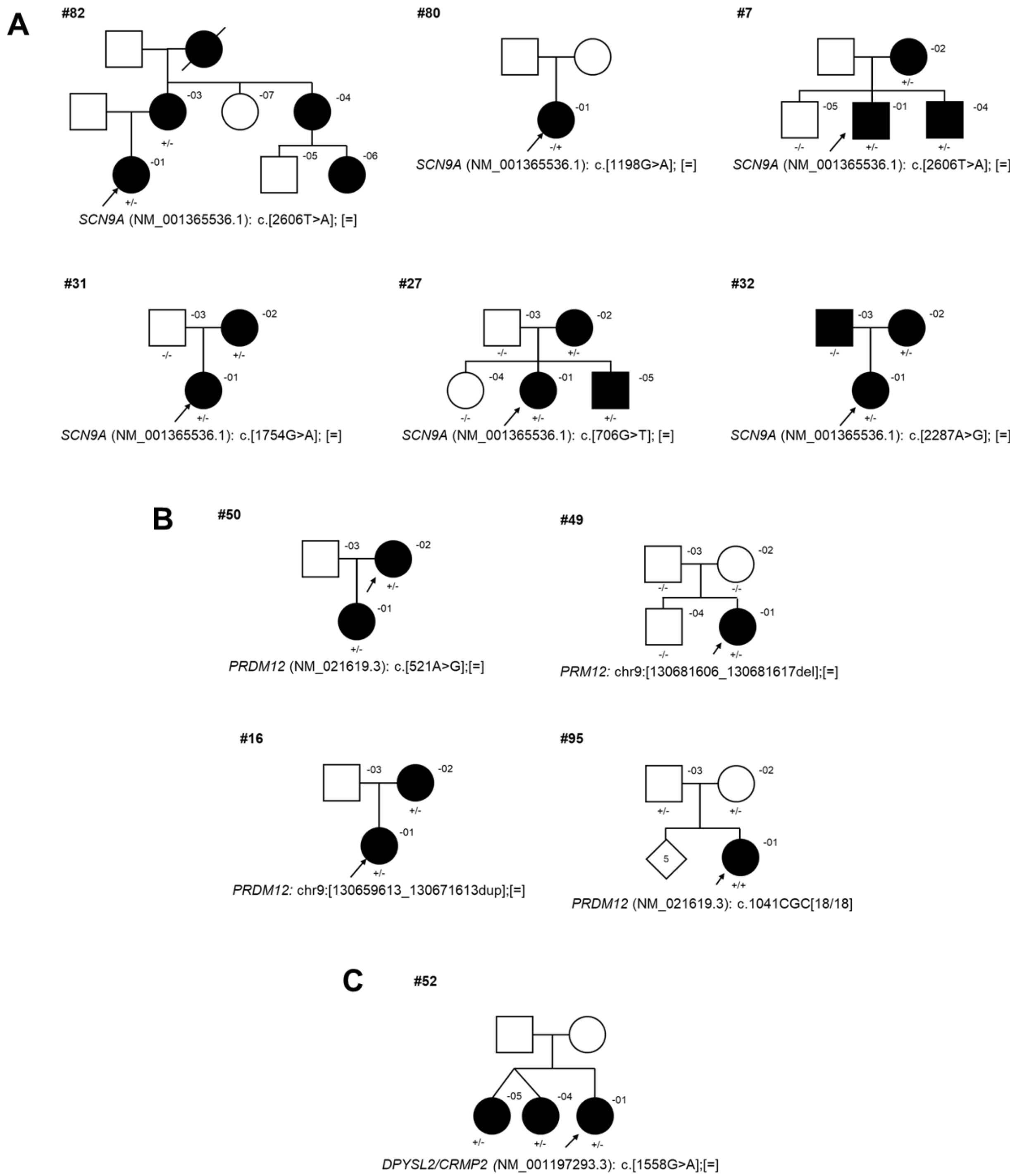

Supplemental Table 1. Overlap between this study's candidate genes and genes/loci discussed in the pain/neuropathy GWAS review by Li et al. [33]. For each overlapping gene, the table notes how the gene is prioritized in this manuscript (e.g., Table 3 category and/or inclusion in the Table 4 SKAT-O gene set) and describes where/how the gene appears in the review (table entry vs narrative; associated phenotype context). This overlap analysis is provided to contextualize rare-variant candidates with common-variant literature and does not imply that the named gene is the causal gene at a GWAS locus.

**Gene**	**Categorization**	**Notable characteristics**
SCN2A	Strong candidate (Table 3); also included in the SKAT-O gene set (Table 4; p-value listed as N/A)	Direct common-variant GWAS/functional-expression link to a sodium channel gene; conceptually aligned with erythromelalgia biology
SCN7A	Moderate candidate (Table 3)	Neuropathy GWAS overlap
EPHA4	Moderate candidate (Table 3)	Notable because it's shown as a repeated locus across distinct pain phenotypes
ADAMTS2	Strong candidate (Table 3)	Points toward a potential neuropathy-related signal (but presented as "suggestive"/not emphasized as replicated in the review narrative)
LAMB3	Strong candidate (Table 3)	Connects more to treatment response / analgesia

Supplemental Table 2. Rare-variant gene-based association testing for housekeeping genes. To control for potential batch effects and systematic bias, two sets of "housekeeping" genes were selected from a published database [18] using a random number generator. For each gene, the table summarizes the number of qualifying variants included (SKAT-O markers), and the uncorrected SKAT-O p-value. SKAT-O was run using a logistic/binary model with Beta(1,25) variant weighting, no covariates, and a coding-variant set definition collapsed by gene; analyses compared 35 affected individuals with 62 matched controls.

**Set**	**Gene**	**SKAT-O markers**	***p*-value (Uncorrected)**	**Set**	**Gene**	**SKAT-O markers**	***p*-value (Uncorrected)**
1	*LCOR*	6	0.46228748	2	*SMARCAL1*	4	0.07635872
1	*ZC3H13*	4	0.40553538	2	*SDAD1*	7	0.19758896
1	*AXIN1*	6	0.70883267	2	*SCRIB*	8	0.31430134
1	*MAZ*	9	0.72877921	2	*GALNS*	4	0.4052064
1	*PHKB*	5	0.57533351	2	*AARS1*	4	0.40531356
1	*FTO*	4	0.40561792	2	*FTO*	4	0.40563877
1	*AARS1*	4	0.40563255	2	*BAZ1B*	4	0.40576462
1	*GALNS*	4	0.40552702	2	*ZC3H13*	4	0.40614549
1	*ZNF91*	6	0.71613389	2	*PI4KA*	4	0.44273587
1	*SMARCAL1*	4	0.07429943	2	*LCOR*	6	0.45270935
1	*PI4KA*	4	0.4423192	2	*PHKB*	5	0.57532659
1	*HTT*	19	0.64513486	2	*HTT*	19	0.64481303
1	*SDAD1*	7	0.19445782	2	*AXIN1*	6	0.70825392
1	*BAZ1B*	4	0.40535789	2	*ZNF91*	6	0.71788249
1	*CNOT4*	4	0.8322901	2	*MAZ*	9	0.72873473
1	*SCRIB*	8	0.31430182	2	*ZXDB*	5	0.79513764
1	*ZXDB*	5	0.79369224	2	*CNOT4*	4	0.83166148

Abbreviations: SKAT-O, Sequence Kernel Association Test - Optimal
